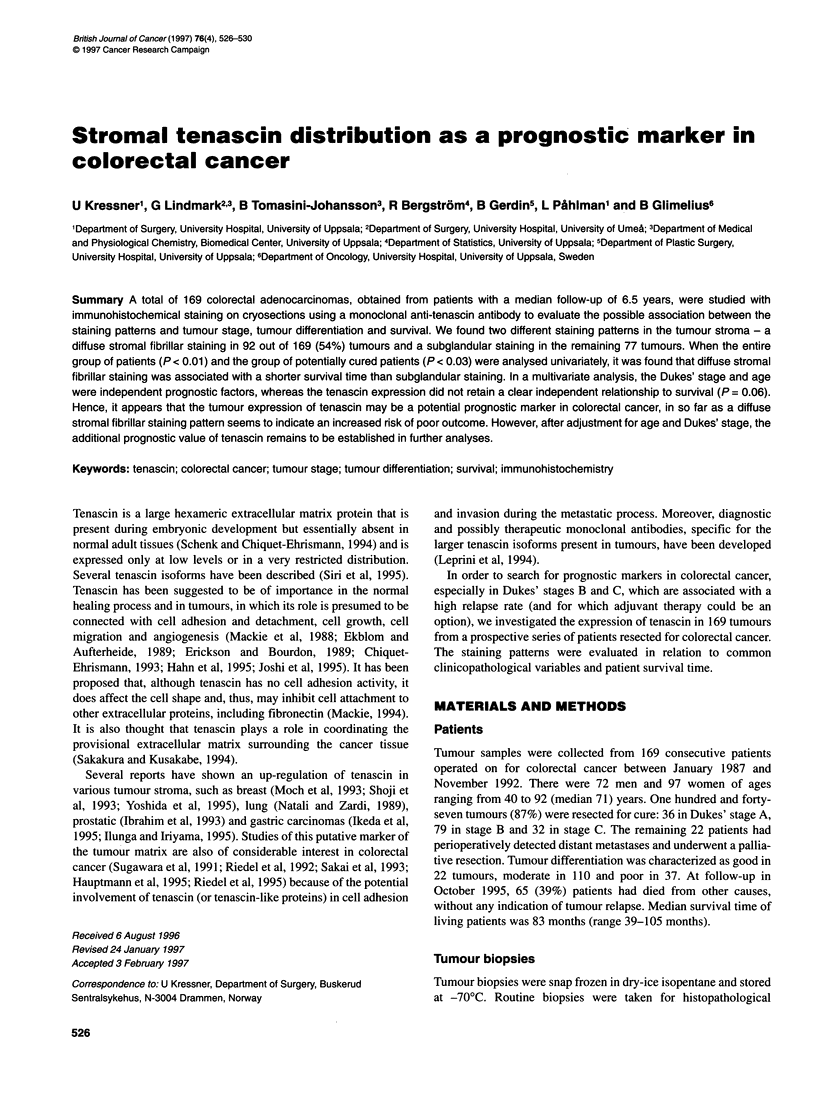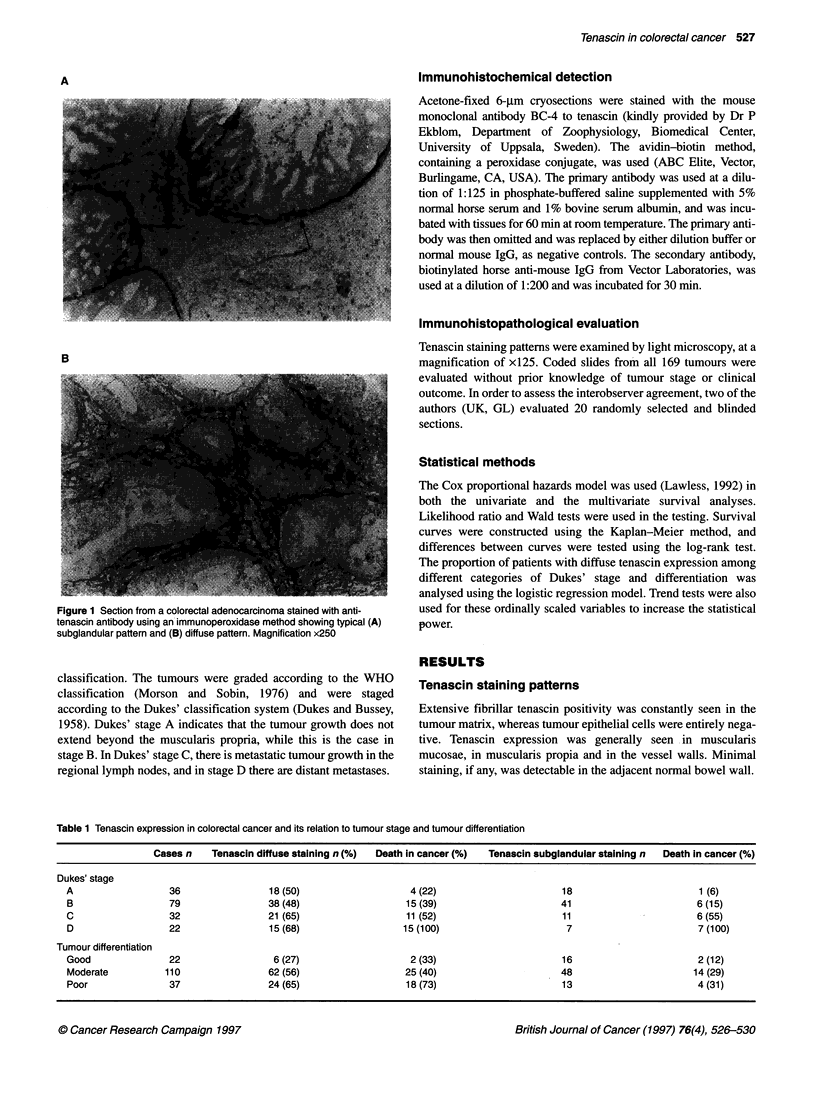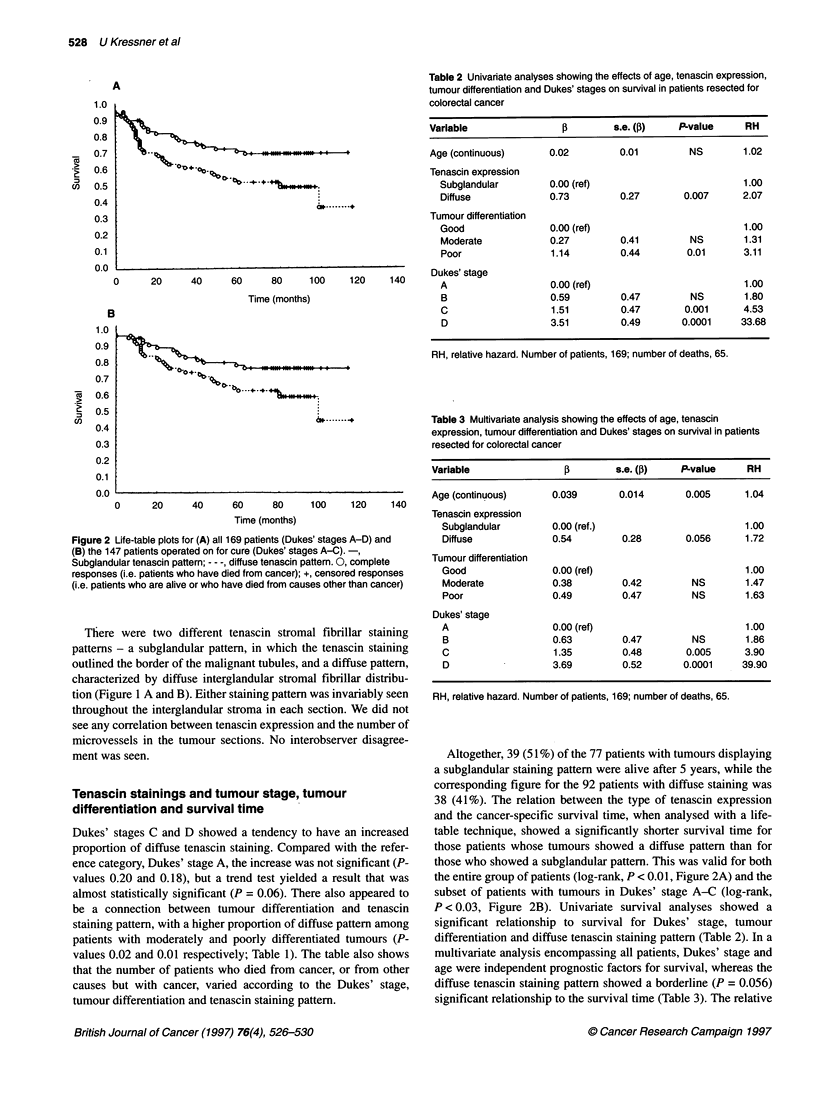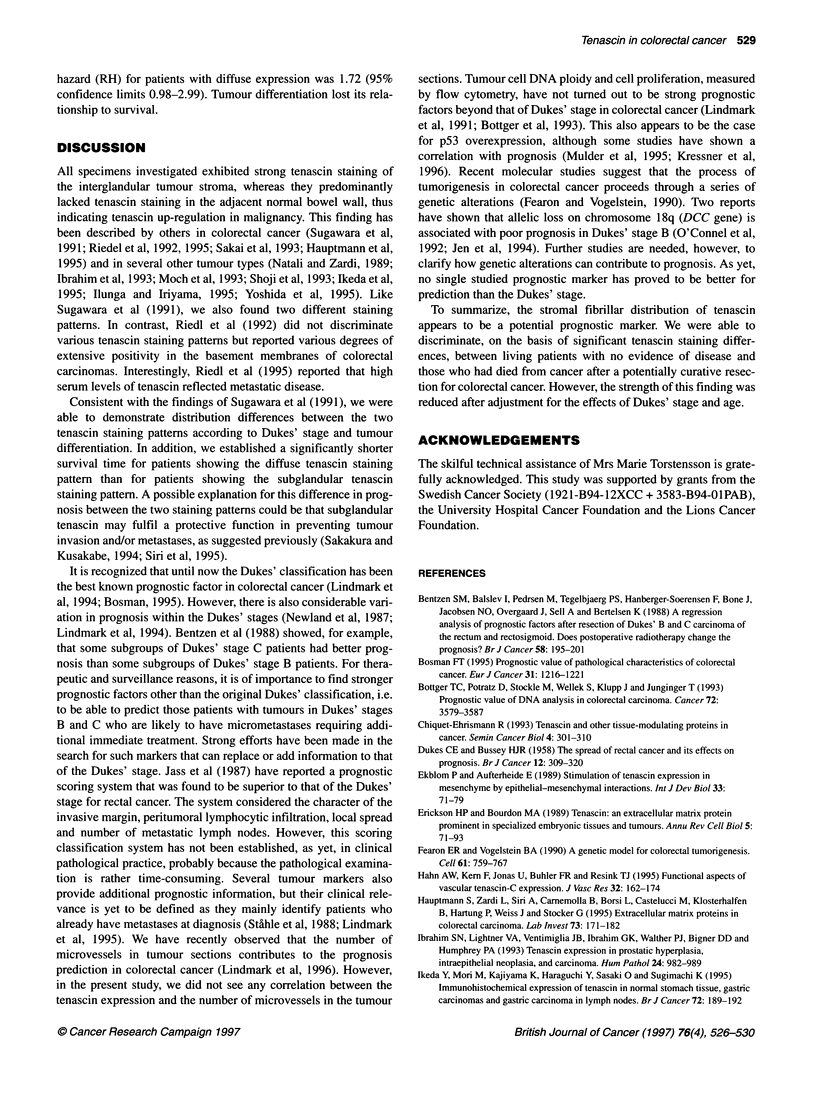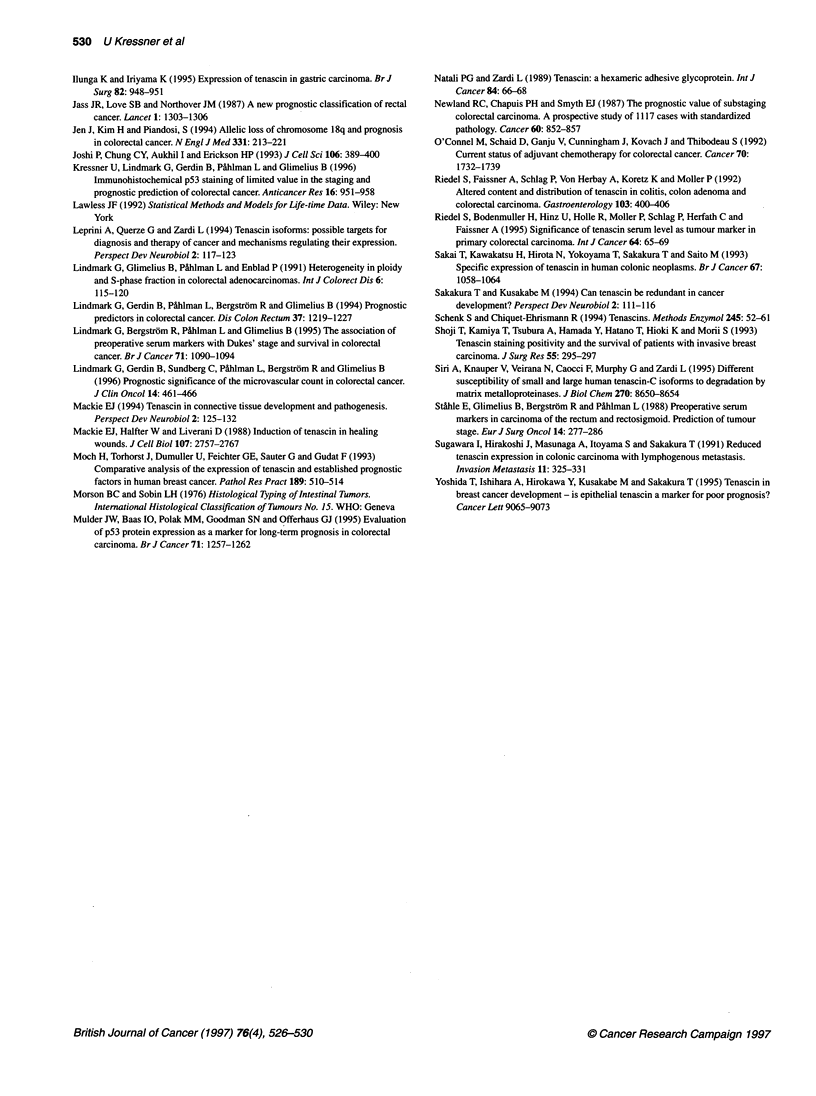# Stromal tenascin distribution as a prognostic marker in colorectal cancer.

**DOI:** 10.1038/bjc.1997.419

**Published:** 1997

**Authors:** U. Kressner, G. Lindmark, B. Tomasini-Johansson, R. BergstrÃ¶m, B. Gerdin, L. PÃ¥hlman, B. Glimelius

**Affiliations:** Department of Surgery, University Hospital, University of Uppsala, Sweden.

## Abstract

**Images:**


*British Journal of Cancer* (1997) **76**(4), 526–530
© 1997 Cancer Research Campaign

### Stromal tenascin distribution as a prognostic marker in colorectal cancer

**U Kressner[1], G Lindmark[2,3], B Tomasini-Johansson[3], R Bergström[4], B Gerdin[5], L Påhlman[1] and B Glimelius[6]**

[1]Department of Surgery, University Hospital, University of Uppsala; [2]Department of Surgery, University Hospital, University of Umeå; [3]Department of Medical and Physiological Chemistry, Biomedical Center, University of Uppsala; [4]Department of Statistics, University of Uppsala; [5]Department of Plastic Surgery, University Hospital, University of Uppsala; [6]Department of Oncology, University Hospital, University of Uppsala, Sweden

**Summary** A total of 169 colorectal adenocarcinomas, obtained from patients with a median follow-up of 6.5 years, were studied with immunohistochemical staining on cryosections using a monoclonal anti-tenascin antibody to evaluate the possible association between the staining patterns and tumour stage, tumour differentiation and survival. We found two different staining patterns in the tumour stroma – a diffuse stromal fibrillar staining in 92 out of 169 (54%) tumours and a subglandular staining in the remaining 77 tumours. When the entire group of patients ($P < 0.01$) and the group of potentially cured patients ($P < 0.03$) were analysed univariately, it was found that diffuse stromal fibrillar staining was associated with a shorter survival time than subglandular staining. In a multivariate analysis, the Dukes' stage and age were independent prognostic factors, whereas the tenascin expression did not retain a clear independent relationship to survival ($P = 0.06$). Hence, it appears that the tumour expression of tenascin may be a potential prognostic marker in colorectal cancer, in so far as a diffuse stromal fibrillar staining pattern seems to indicate an increased risk of poor outcome. However, after adjustment for age and Dukes' stage, the additional prognostic value of tenascin remains to be established in further analyses.

**Keywords:** tenascin; colorectal cancer; tumour stage; tumour differentiation; survival; immunohistochemistry

Tenascin is a large hexameric extracellular matrix protein that is present during embryonic development but essentially absent in normal adult tissues (Schenk and Chiquet-Ehrismann, 1994) and is expressed only at low levels or in a very restricted distribution. Several tenascin isoforms have been described (Siri et al, 1995). Tenascin has been suggested to be of importance in the normal healing process and in tumours, in which its role is presumed to be connected with cell adhesion and detachment, cell growth, cell migration and angiogenesis (Mackie et al, 1988; Ekblom and Aufterheide, 1989; Erickson and Bourdon, 1989; Chiquet-Ehrismann, 1993; Hahn et al, 1995; Joshi et al, 1995). It has been proposed that, although tenascin has no cell adhesion activity, it does affect the cell shape and, thus, may inhibit cell attachment to other extracellular proteins, including fibronectin (Mackie, 1994). It is also thought that tenascin plays a role in coordinating the provisional extracellular matrix surrounding the cancer tissue (Sakakura and Kusakabe, 1994).

Several reports have shown an up-regulation of tenascin in various tumour stroma, such as breast (Moch et al, 1993; Shoji et al, 1993; Yoshida et al, 1995), lung (Natali and Zardi, 1989), prostatic (Ibrahim et al, 1993) and gastric carcinomas (Ikeda et al, 1995; Ilunga and Iriyama, 1995). Studies of this putative marker of the tumour matrix are also of considerable interest in colorectal cancer (Sugawara et al, 1991; Riedel et al, 1992; Sakai et al, 1993; Hauptmann et al, 1995; Riedel et al, 1995) because of the potential involvement of tenascin (or tenascin-like proteins) in cell adhesion and invasion during the metastatic process. Moreover, diagnostic and possibly therapeutic monoclonal antibodies, specific for the larger tenascin isoforms present in tumours, have been developed (Leprini et al, 1994).

In order to search for prognostic markers in colorectal cancer, especially in Dukes' stages B and C, which are associated with a high relapse rate (and for which adjuvant therapy could be an option), we investigated the expression of tenascin in 169 tumours from a prospective series of patients resected for colorectal cancer. The staining patterns were evaluated in relation to common clinicopathological variables and patient survival time.

### MATERIALS AND METHODS

### Patients

Tumour samples were collected from 169 consecutive patients operated on for colorectal cancer between January 1987 and November 1992. There were 72 men and 97 women of ages ranging from 40 to 92 (median 71) years. One hundred and forty-seven tumours (87%) were resected for cure: 36 in Dukes' stage A, 79 in stage B and 32 in stage C. The remaining 22 patients had perioperatively detected distant metastases and underwent a palliative resection. Tumour differentiation was characterized as good in 22 tumours, moderate in 110 and poor in 37. At follow-up in October 1995, 65 (39%) patients had died from other causes, without any indication of tumour relapse. Median survival time of living patients was 83 months (range 39–105 months).

### Tumour biopsies

Tumour biopsies were snap frozen in dry-ice isopentane and stored at –70°C. Routine biopsies were taken for histopathological

*Received 6 August 1996*
*Revised 24 January 1997*
*Accepted 3 February 1997*

*Correspondence to:* U Kressner, Department of Surgery, Buskerud Sentralsykehus, N-3004 Drammen, Norway

**Figure 1** Section from a colorectal adenocarcinoma stained with anti-tenascin antibody using an immunoperoxidase method showing typical (**A**) subglandular pattern and (**B**) diffuse pattern. Magnification ×250

classification. The tumours were graded according to the WHO classification (Morson and Sobin, 1976) and were staged according to the Dukes' classification system (Dukes and Bussey, 1958). Dukes' stage A indicates that the tumour growth does not extend beyond the muscularis propria, while this is the case in stage B. In Dukes' stage C, there is metastatic tumour growth in the regional lymph nodes, and in stage D there are distant metastases.

### Immunohistochemical detection

Acetone-fixed 6-μm cryosections were stained with the mouse monoclonal antibody BC-4 to tenascin (kindly provided by Dr P Ekblom, Department of Zoophysiology, Biomedical Center, University of Uppsala, Sweden). The avidin–biotin method, containing a peroxidase conjugate, was used (ABC Elite, Vector, Burlingame, CA, USA). The primary antibody was used at a dilution of 1:125 in phosphate-buffered saline supplemented with 5% normal horse serum and 1% bovine serum albumin, and was incubated with tissues for 60 min at room temperature. The primary antibody was then omitted and was replaced by either dilution buffer or normal mouse IgG, as negative controls. The secondary antibody, biotinylated horse anti-mouse IgG from Vector Laboratories, was used at a dilution of 1:200 and was incubated for 30 min.

### Immunohistopathological evaluation

Tenascin staining patterns were examined by light microscopy, at a magnification of ×125. Coded slides from all 169 tumours were evaluated without prior knowledge of tumour stage or clinical outcome. In order to assess the interobserver agreement, two of the authors (UK, GL) evaluated 20 randomly selected and blinded sections.

### Statistical methods

The Cox proportional hazards model was used (Lawless, 1992) in both the univariate and the multivariate survival analyses. Likelihood ratio and Wald tests were used in the testing. Survival curves were constructed using the Kaplan–Meier method, and differences between curves were tested using the log-rank test. The proportion of patients with diffuse tenascin expression among different categories of Dukes' stage and differentiation was analysed using the logistic regression model. Trend tests were also used for these ordinally scaled variables to increase the statistical power.

### RESULTS

### Tenascin staining patterns

Extensive fibrillar tenascin positivity was constantly seen in the tumour matrix, whereas tumour epithelial cells were entirely negative. Tenascin expression was generally seen in muscularis mucosae, in muscularis propia and in the vessel walls. Minimal staining, if any, was detectable in the adjacent normal bowel wall.

**Table 1** Tenascin expression in colorectal cancer and its relation to tumour stage and tumour differentiation

| | Cases *n* | Tenascin diffuse staining *n* (%) | Death in cancer (%) | Tenascin subglandular staining *n* | Death in cancer (%) |
|---|---|---|---|---|---|
| Dukes' stage | | | | | |
| A | 36 | 18 (50) | 4 (22) | 18 | 1 (6) |
| B | 79 | 38 (48) | 15 (39) | 41 | 6 (15) |
| C | 32 | 21 (65) | 11 (52) | 11 | 6 (55) |
| D | 22 | 15 (68) | 15 (100) | 7 | 7 (100) |
| Tumour differentiation | | | | | |
| Good | 22 | 6 (27) | 2 (33) | 16 | 2 (12) |
| Moderate | 110 | 62 (56) | 25 (40) | 48 | 14 (29) |
| Poor | 37 | 24 (65) | 18 (73) | 13 | 4 (31) |

© Cancer Research Campaign 1997 

**Figure 2** Life-table plots for (**A**) all 169 patients (Dukes' stages A–D) and (**B**) the 147 patients operated on for cure (Dukes' stages A–C). —, Subglandular tenascin pattern; - - -, diffuse tenascin pattern. O, complete responses (i.e. patients who have died from cancer); +, censored responses (i.e. patients who are alive or who have died from causes other than cancer)

There were two different tenascin stromal fibrillar staining patterns – a subglandular pattern, in which the tenascin staining outlined the border of the malignant tubules, and a diffuse pattern, characterized by diffuse interglandular stromal fibrillar distribution (Figure 1 A and B). Either staining pattern was invariably seen throughout the interglandular stroma in each section. We did not see any correlation between tenascin expression and the number of microvessels in the tumour sections. No interobserver disagreement was seen.

### Tenascin stainings and tumour stage, tumour differentiation and survival time

Dukes' stages C and D showed a tendency to have an increased proportion of diffuse tenascin staining. Compared with the reference category, Dukes' stage A, the increase was not significant (*P*-values 0.20 and 0.18), but a trend test yielded a result that was almost statistically significant ($P = 0.06$). There also appeared to be a connection between tumour differentiation and tenascin staining pattern, with a higher proportion of diffuse pattern among patients with moderately and poorly differentiated tumours (*P*-values 0.02 and 0.01 respectively; Table 1). The table also shows that the number of patients who died from cancer, or from other causes but with cancer, varied according to the Dukes' stage, tumour differentiation and tenascin staining pattern.

**Table 2** Univariate analyses showing the effects of age, tenascin expression, tumour differentiation and Dukes' stages on survival in patients resected for colorectal cancer

| Variable | β | s.e. (β) | *P*-value | RH |
|---|---|---|---|---|
| Age (continuous) | 0.02 | 0.01 | NS | 1.02 |
| Tenascin expression | | | | |
| Subglandular | 0.00 (ref) | | | 1.00 |
| Diffuse | 0.73 | 0.27 | 0.007 | 2.07 |
| Tumour differentiation | | | | |
| Good | 0.00 (ref) | | | 1.00 |
| Moderate | 0.27 | 0.41 | NS | 1.31 |
| Poor | 1.14 | 0.44 | 0.01 | 3.11 |
| Dukes' stage | | | | |
| A | 0.00 (ref) | | | 1.00 |
| B | 0.59 | 0.47 | NS | 1.80 |
| C | 1.51 | 0.47 | 0.001 | 4.53 |
| D | 3.51 | 0.49 | 0.0001 | 33.68 |

RH, relative hazard. Number of patients, 169; number of deaths, 65.

**Table 3** Multivariate analysis showing the effects of age, tenascin expression, tumour differentiation and Dukes' stages on survival in patients resected for colorectal cancer

| Variable | β | s.e. (β) | *P*-value | RH |
|---|---|---|---|---|
| Age (continuous) | 0.039 | 0.014 | 0.005 | 1.04 |
| Tenascin expression | | | | |
| Subglandular | 0.00 (ref.) | | | 1.00 |
| Diffuse | 0.54 | 0.28 | 0.056 | 1.72 |
| Tumour differentiation | | | | |
| Good | 0.00 (ref) | | | 1.00 |
| Moderate | 0.38 | 0.42 | NS | 1.47 |
| Poor | 0.49 | 0.47 | NS | 1.63 |
| Dukes' stage | | | | |
| A | 0.00 (ref) | | | 1.00 |
| B | 0.63 | 0.47 | NS | 1.86 |
| C | 1.35 | 0.48 | 0.005 | 3.90 |
| D | 3.69 | 0.52 | 0.0001 | 39.90 |

RH, relative hazard. Number of patients, 169; number of deaths, 65.

Altogether, 39 (51%) of the 77 patients with tumours displaying a subglandular staining pattern were alive after 5 years, while the corresponding figure for the 92 patients with diffuse staining was 38 (41%). The relation between the type of tenascin expression and the cancer-specific survival time, when analysed with a life-table technique, showed a significantly shorter survival time for those patients whose tumours showed a diffuse pattern than for those who showed a subglandular pattern. This was valid for both the entire group of patients (log-rank, $P < 0.01$, Figure 2A) and the subset of patients with tumours in Dukes' stage A–C (log-rank, $P < 0.03$, Figure 2B). Univariate survival analyses showed a significant relationship to survival for Dukes' stage, tumour differentiation and diffuse tenascin staining pattern (Table 2). In a multivariate analysis encompassing all patients, Dukes' stage and age were independent prognostic factors for survival, whereas the diffuse tenascin staining pattern showed a borderline ($P = 0.056$) significant relationship to the survival time (Table 3). The relative

 © Cancer Research Campaign 1997

hazard (RH) for patients with diffuse expression was 1.72 (95% confidence limits 0.98–2.99). Tumour differentiation lost its relationship to survival.

### DISCUSSION

All specimens investigated exhibited strong tenascin staining of the interglandular tumour stroma, whereas they predominantly lacked tenascin staining in the adjacent normal bowel wall, thus indicating tenascin up-regulation in malignancy. This finding has been described by others in colorectal cancer (Sugawara et al, 1991; Riedel et al, 1992, 1995; Sakai et al, 1993; Hauptmann et al, 1995) and in several other tumour types (Natali and Zardi, 1989; Ibrahim et al, 1993; Moch et al, 1993; Shoji et al, 1993; Ikeda et al, 1995; Ilunga and Iriyama, 1995; Yoshida et al, 1995). Like Sugawara et al (1991), we also found two different staining patterns. In contrast, Riedl et al (1992) did not discriminate various tenascin staining patterns but reported various degrees of extensive positivity in the basement membranes of colorectal carcinomas. Interestingly, Riedl et al (1995) reported that high serum levels of tenascin reflected metastatic disease.

Consistent with the findings of Sugawara et al (1991), we were able to demonstrate distribution differences between the two tenascin staining patterns according to Dukes' stage and tumour differentiation. In addition, we established a significantly shorter survival time for patients showing the diffuse tenascin staining pattern than for patients showing the subglandular tenascin staining pattern. A possible explanation for this difference in prognosis between the two staining patterns could be that subglandular tenascin may fulfil a protective function in preventing tumour invasion and/or metastases, as suggested previously (Sakakura and Kusakabe, 1994; Siri et al, 1995).

It is recognized that until now the Dukes' classification has been the best known prognostic factor in colorectal cancer (Lindmark et al, 1994; Bosman, 1995). However, there is also considerable variation in prognosis within the Dukes' stages (Newland et al, 1987; Lindmark et al, 1994). Bentzen et al (1988) showed, for example, that some subgroups of Dukes' stage C patients had better prognosis than some subgroups of Dukes' stage B patients. For therapeutic and surveillance reasons, it is of importance to find stronger prognostic factors other than the original Dukes' classification, i.e. to be able to predict those patients with tumours in Dukes' stages B and C who are likely to have micrometastases requiring additional immediate treatment. Strong efforts have been made in the search for such markers that can replace or add information to that of the Dukes' stage. Jass et al (1987) have reported a prognostic scoring system that was found to be superior to that of the Dukes' stage for rectal cancer. The system considered the character of the invasive margin, peritumoral lymphocytic infiltration, local spread and number of metastatic lymph nodes. However, this scoring classification system has not been established, as yet, in clinical pathological practice, probably because the pathological examination is rather time-consuming. Several tumour markers also provide additional prognostic information, but their clinical relevance is yet to be defined as they mainly identify patients who already have metastases at diagnosis (Ståhle et al, 1988; Lindmark et al, 1995). We have recently observed that the number of microvessels in tumour sections contributes to the prognosis prediction in colorectal cancer (Lindmark et al, 1996). However, in the present study, we did not see any correlation between the tenascin expression and the number of microvessels in the tumour sections. Tumour cell DNA ploidy and cell proliferation, measured by flow cytometry, have not turned out to be strong prognostic factors beyond that of Dukes' stage in colorectal cancer (Lindmark et al, 1991; Bottger et al, 1993). This also appears to be the case for p53 overexpression, although some studies have shown a correlation with prognosis (Mulder et al, 1995; Kressner et al, 1996). Recent molecular studies suggest that the process of tumorigenesis in colorectal cancer proceeds through a series of genetic alterations (Fearon and Vogelstein, 1990). Two reports have shown that allelic loss on chromosome 18q (*DCC* gene) is associated with poor prognosis in Dukes' stage B (O'Connel et al, 1992; Jen et al, 1994). Further studies are needed, however, to clarify how genetic alterations can contribute to prognosis. As yet, no single studied prognostic marker has proved to be better for prediction than the Dukes' stage.

To summarize, the stromal fibrillar distribution of tenascin appears to be a potential prognostic marker. We were able to discriminate, on the basis of significant tenascin staining differences, between living patients with no evidence of disease and those who had died from cancer after a potentially curative resection for colorectal cancer. However, the strength of this finding was reduced after adjustment for the effects of Dukes' stage and age.

### ACKNOWLEDGEMENTS

The skilful technical assistance of Mrs Marie Torstensson is gratefully acknowledged. This study was supported by grants from the Swedish Cancer Society (1921-B94-12XCC + 3583-B94-01PAB), the University Hospital Cancer Foundation and the Lions Cancer Foundation.

### REFERENCES

Bentzen SM, Balslev I, Pedrsen M, Tegelbjaerg PS, Hanberger-Soerensen F, Bone J, Jacobsen NO, Overgaard J, Sell A and Bertelsen K (1988) A regression analysis of prognostic factors after resection of Dukes' B and C carcinoma of the rectum and rectosigmoid. Does postoperative radiotherapy change the prognosis? *Br J Cancer* **58**: 195–201

Bosman FT (1995) Prognostic value of pathological characteristics of colorectal cancer. *Eur J Cancer* **31**: 1216–1221

Bottger TC, Potratz D, Stockle M, Wellek S, Klupp J and Junginger T (1993) Prognostic value of DNA analysis in colorectal carcinoma. *Cancer* **72**: 3579–3587

Chiquet-Ehrismann R (1993) Tenascin and other tissue-modulating proteins in cancer. *Semin Cancer Biol* **4**: 301–310

Dukes CE and Bussey HJR (1958) The spread of rectal cancer and its effects on prognosis. *Br J Cancer* **12**: 309–320

Ekblom P and Aufterheide E (1989) Stimulation of tenascin expression in mesenchyme by epithelial–mesenchymal interactions. *Int J Dev Biol* **33**: 71–79

Erickson HP and Bourdon MA (1989) Tenascin: an extracellular matrix protein prominent in specialized embryonic tissues and tumours. *Annu Rev Cell Biol* **5**: 71–93

Fearon ER and Vogelstein BA (1990) A genetic model for colorectal tumorigenesis. *Cell* **61**: 759–767

Hahn AW, Kern F, Jonas U, Buhler FR and Resink TJ (1995) Functional aspects of vascular tenascin-C expression. *J Vasc Res* **32**: 162–174

Hauptmann S, Zardi L, Siri A, Carnemolla B, Borsi L, Castelucci M, Klosterhalfen B, Hartung P, Weiss J and Stocker G (1995) Extracellular matrix proteins in colorectal carcinoma. *Lab Invest* **73**: 171–182

Ibrahim SN, Lightner VA, Ventimiglia JB, Ibrahim GK, Walther PJ, Bigner DD and Humphrey PA (1993) Tenascin expression in prostatic hyperplasia, intraepithelial neoplasia, and carcinoma. *Hum Pathol* **24**: 982–989

Ikeda Y, Mori M, Kajiyama K, Haraguchi Y, Sasaki O and Sugimachi K (1995) Immunohistochemical expression of tenascin in normal stomach tissue, gastric carcinomas and gastric carcinoma in lymph nodes. *Br J Cancer* **72**: 189–192

© Cancer Research Campaign 1997 

Ilunga K and Iriyama K (1995) Expression of tenascin in gastric carcinoma. *Br J Surg* **82**: 948–951

Jass JR, Love SB and Northover JM (1987) A new prognostic classification of rectal cancer. *Lancet* **1**: 1303–1306

Jen J, Kim H and Piandosi, S (1994) Allelic loss of chromosome 18q and prognosis in colorectal cancer. *N Engl J Med* **331**: 213–221

Joshi P, Chung CY, Aukhil I and Erickson HP (1993) *J Cell Sci* **106**: 389–400

Kressner U, Lindmark G, Gerdin B, Påhlman L and Glimelius B (1996) Immunohistochemical p53 staining of limited value in the staging and prognostic prediction of colorectal cancer. *Anticancer Res* **16**: 951–958

Lawless JF (1992) *Statistical Methods and Models for Life-time Data.* Wiley: New York

Leprini A, Querze G and Zardi L (1994) Tenascin isoforms: possible targets for diagnosis and therapy of cancer and mechanisms regulating their expression. *Perspect Dev Neurobiol* **2**: 117–123

Lindmark G, Glimelius B, Påhlman L and Enblad P (1991) Heterogeneity in ploidy and S-phase fraction in colorectal adenocarcinomas. *Int J Colorect Dis* **6**: 115–120

Lindmark G, Gerdin B, Påhlman L, Bergström R and Glimelius B (1994) Prognostic predictors in colorectal cancer. *Dis Colon Rectum* **37**: 1219–1227

Lindmark G, Bergström R, Påhlman L and Glimelius B (1995) The association of preoperative serum markers with Dukes' stage and survival in colorectal cancer. *Br J Cancer* **71**: 1090–1094

Lindmark G, Gerdin B, Sundberg C, Påhlman L, Bergström R and Glimelius B (1996) Prognostic significance of the microvascular count in colorectal cancer. *J Clin Oncol* **14**: 461–466

Mackie EJ (1994) Tenascin in connective tissue development and pathogenesis. *Perspect Dev Neurobiol* **2**: 125–132

Mackie EJ, Halfter W and Liverani D (1988) Induction of tenascin in healing wounds. *J Cell Biol* **107**: 2757–2767

Moch H, Torhorst J, Dumuller U, Feichter GE, Sauter G and Gudat F (1993) Comparative analysis of the expression of tenascin and established prognostic factors in human breast cancer. *Pathol Res Pract* **189**: 510–514

Morson BC and Sobin LH (1976) *Histological Typing of Intestinal Tumors. International Histological Classification of Tumours No. 15.* WHO: Geneva

Mulder JW, Baas IO, Polak MM, Goodman SN and Offerhaus GJ (1995) Evaluation of p53 protein expression as a marker for long-term prognosis in colorectal carcinoma. *Br J Cancer* **71**: 1257–1262

Natali PG and Zardi L (1989) Tenascin: a hexameric adhesive glycoprotein. *Int J Cancer* **84**: 66–68

Newland RC, Chapuis PH and Smyth EJ (1987) The prognostic value of substaging colorectal carcinoma. A prospective study of 1117 cases with standardized pathology. *Cancer* **60**: 852–857

O'Connel M, Schaid D, Ganju V, Cunningham J, Kovach J and Thibodeau S (1992) Current status of adjuvant chemotherapy for colorectal cancer. *Cancer* **70**: 1732–1739

Riedel S, Faissner A, Schlag P, Von Herbay A, Koretz K and Moller P (1992) Altered content and distribution of tenascin in colitis, colon adenoma and colorectal carcinoma. *Gastroenterology* **103**: 400–406

Riedel S, Bodenmuller H, Hinz U, Holle R, Moller P, Schlag P, Herfath C and Faissner A (1995) Significance of tenascin serum level as tumour marker in primary colorectal carcinoma. *Int J Cancer* **64**: 65–69

Sakai T, Kawakatsu H, Hirota N, Yokoyama T, Sakakura T and Saito M (1993) Specific expression of tenascin in human colonic neoplasms. *Br J Cancer* **67**: 1058–1064

Sakakura T and Kusakabe M (1994) Can tenascin be redundant in cancer development? *Perspect Dev Neurobiol* **2**: 111–116

Schenk S and Chiquet-Ehrismann R (1994) Tenascins. *Methods Enzymol* **245**: 52–61

Shoji T, Kamiya T, Tsubura A, Hamada Y, Hatano T, Hioki K and Morii S (1993) Tenascin staining positivity and the survival of patients with invasive breast carcinoma. *J Surg Res* **55**: 295–297

Siri A, Knauper V, Veirana N, Caocci F, Murphy G and Zardi L (1995) Different susceptibility of small and large human tenascin-C isoforms to degradation by matrix metalloproteinases. *J Biol Chem* **270**: 8650–8654

Ståhle E, Glimelius B, Bergström R and Påhlman L (1988) Preoperative serum markers in carcinoma of the rectum and rectosigmoid. Prediction of tumour stage. *Eur J Surg Oncol* **14**: 277–286

Sugawara I, Hirakoshi J, Masunaga A, Itoyama S and Sakakura T (1991) Reduced tenascin expression in colonic carcinoma with lymphogenous metastasis. *Invasion Metastasis* **11**: 325–331

Yoshida T, Ishihara A, Hirokawa Y, Kusakabe M and Sakakura T (1995) Tenascin in breast cancer development – is epithelial tenascin a marker for poor prognosis? *Cancer Lett* 9065–9073

© Cancer Research Campaign 1997